# The economic status of older people’s households in urban and rural settings in Peru, Mexico and China: a 10/66 INDEP study cross-sectional survey

**DOI:** 10.1186/s40064-016-1913-2

**Published:** 2016-03-02

**Authors:** Martin J. Prince, Peter Lloyd-Sherlock, Mariella Guerra, Yueqin Huang, Ana Luisa Sosa, Richard Uwakwe, Isaac Acosta, Zhaorui Liu, Sara Gallardo, Maelenn Guerchet, Rosie Mayston, Veronica Montes de Oca, Hong Wang, Peter Ezeah

**Affiliations:** Health Service and Population Research Department, Institute of Psychiatry, Psychology and Neuroscience, King’s College London, P060, De Crespigny Park, London, SE5 8AF UK; School of Development Studies, University of East Anglia, Norwich, NR4 7TJ UK; Psychogeriatric Unit, National Institute of Mental Health “Honorio Delgado Hideyo Noguchi”, Jr. Eloy Espinoza 709, Urb. Palao, San Martin de Porres, Lima, Peru; Peking University, Institute of Mental Health, No. 51 Hua Yuan Bei Road, Haidian District, Beijing, 100191 People’s Republic of China; National Institute of Neurology and Neurosurgery of Mexico, Universidad Nacional Autónoma de México, Insurgentes Sur 3877, 14269 Mexico City, Mexico; Nnamdi Azikiwe University Teaching Hospital, Nnewi, Anambra State Nigeria; Instituto de la Memoria, Depresión y Enfermedades de Riesgo (IMEDER), Av. Constructores 1230, La Molina, Lima Peru; Instituto de Investigaciones Sociales, Universidad Nacional Autónoma de México, Mexico City, Mexico; Department of Sociology/Anthropology, Nnamdi Azikiwe University Awka, PMB 5025, Awka, Anambra State Nigeria

**Keywords:** Ageing, Developing countries, Mexico, Peru, China, Pensions, Economic status

## Abstract

Few data are available from middle income countries regarding economic circumstances of households in which older people live. Many such settings have experienced rapid demographic, social and economic change, alongside increasing pension coverage. Population-based household surveys in rural and urban catchment areas in Peru, Mexico and China. Participating households were selected from all households with older residents. Descriptive analyses were weighted back for sampling fractions and non-response. Household income and consumption were estimated from a household key informant interview. 877 Household interviews (3177 residents). Response rate 68 %. Household income and consumption correlated plausibly with other economic wellbeing indicators. Household Incomes varied considerably within and between sites. While multigenerational households were the norm, older resident’s incomes accounted for a high proportion of household income, and older people were particularly likely to pool income. Differences in the coverage and value of pensions were a major source of variation in household income among sites. There was a small, consistent inverse association between household pension income and labour force participation of younger adult co-residents. The effect of pension income on older adults’ labour force participation was less clear-cut. Historical linkage of social protection to formal employment may have contributed to profound late-life socioeconomic inequalities. Strategies to formalise the informal economy, alongside increases in the coverage and value of non-contributory pensions and transfers would help to address this problem.

## Background

Population ageing is advancing at an unprecedented pace, particularly in rapidly developing middle income countries in Latin America and Asia (Kinsella and Phillips 2005; National Institute on Aging 2011). This transition poses challenges to governments and societies seeking to assure social protection while maintaining intergenerational equity and fiscal prudence. At the same time, traditional family-based systems of support and care for older people are under threat as fertility rates fall, children migrate away in search of work and economic advancement, and better educated women are less available to take on caring roles. Social protection of older people is promoted first and foremost through an increase in the coverage and value of their pensions. Reforms of health care financing, towards affordable health insurance or health care free at the point of delivery are a further effective insurance against the risks that older people face. As yet, social care to supplement or substitute informal care arrangements is not generally available, other than through private arrangements made by some higher income families in, mainly, urban settings (Prince et al. 2008).

Few detailed data are currently available regarding the economic circumstances of households in which older people live, in societies undergoing rapid demographic, social and economic change. Nationally representative surveys are limited to varying degrees by the restricted coverage of income sources, and the extent to which the income of household members other than older people and their spouses are systematically ascertained (Angrisani and Lee 2011). Simulations of, for example, the poverty reduction potential of social pension programs are limited by the lack of available data on consumption, assets, income pooling, and links between pensions and labour force participation (Gasparini et al. 2010; Olivera and Zuluaga 2014).

The relative benefits of measures of household income and consumption to define households as poor or non-poor are debated in high income countries (Meyer and Sullivan 2012). An important consideration for older people’s households is that consumption patterns can capture the effects of using accumulated wealth and the access to credit that this provides to consume more than is earned (Hurd and Rohwedder 2006). In less developed economies, income measures may underestimate economic utility given the importance of informal jobs, barter arrangements, and, in rural communities, the value of food grown and consumed. Incomes tend to be under-reported, particularly in low income households with diverse sources of income that include government and private transfers (Meyer and Sullivan 2012). Nevertheless, income has been more widely used to assess economic well-being and poverty rates, in part because the collection of comprehensive consumption data can take up a lot of survey time.

The INDEP study (Mayston et al. 2014) is nested within a subset of the 10/66 Dementia Research group’s longitudinal population-based catchment area surveys of older people in Latin America, Asia and Africa (Prince et al. 2007). The ultimate objective is to evaluate the impact of care dependence among older people on household economic and social functioning. The protocol includes a particularly detailed assessment of household income (and sources), consumption, and assets (Mayston et al. 2014). While household selection for the INDEP study was stratified according to the needs for care of older residents, weighting back to the original sampling fractions enables us to generalize findings to all households within the urban and rural catchment areas studied in Peru, Mexico and China. Our aims in these formative analyses are to capture, in detail, the impact of the rapidly evolving macro-policy context in these countries on the economic status of households in which older people live, in the specific INDEP study catchment areas studied. We were particularly interested in the extent and origins of socioeconomic inequalities within and between the INDEP catchment areas, and the salience of older person’s incomes to household finances. Given concerns regarding potential perverse incentives of cash transfers, we set out to estimate the effect of household pension income upon labour force participation of younger and older adult residents. We also sought to explore the comparative validity of measures of household income and consumption as indicators of poverty in these settings. Specifically, we aimed to.

1. Describe the distribution of household income and consumption, and its equity, among older people’s households in INDEP rural and urban catchment areas in Peru, Mexico and China, and the levels of relative and absolute poverty according to widely used criteria.
2. Compare the income levels of younger adults and older adults, in older people’s households, and their propensity to pool that income among household members.
3. Estimate the distribution of household income by source, and the proportion of older people’s household incomes contributed by older adults.
4. Test the hypotheses that household income from pensions is independently inversely associated with individual labour force participation of (a) working age adults, and (b) older adults.
5. Explore the construct validity of measures of household income and consumption through their correlation with other proximal indicators of economic status, and through associations of income-based and consumption-based relative poverty indicators with other indicators of economic utility.

Our analyses add value in a number of ways. We examine different indicators of poverty. We make systematic comparisons across rural and urban sites in three countries with distinct social protection systems. We examine inequalities within the older catchment area populations, building on the existing literature debating the extent and origins of socioeconomic disadvantage among older people, as a unitary class (Kidd and Whitehouse 2009).

## Results

In all, 877 household interviews were completed with an overall response rate of 68 %. Site specific response rates were; urban Peru 55 %, rural Peru 59 %, urban Mexico 86 %, rural Mexico 78 %, urban China 52 %, and rural China 91 %. The 877 households weighted back (accounting for sampling and non-response) to 3390 households from the original incidence wave sample. Information was collected on 3177 individual residents of these older people’s households, weighting back to an estimated 12,669 residents from the original sample.

In all sites the mode was for older people to live in multigenerational households with younger adults, and, often, children under the age of 16 (Table 1). The urban China site stood out as having smaller households, a higher proportion of households where older people lived without younger adults (39.2 %), and a very low proportion of households with co-resident children (3.8 %). In all sites other than urban China, the proportions of all residents of older person’s households who were younger adults were higher than the proportions who were older adults. The proportion of all residents who were older adults was 54.6 % in urban China and proportions ranged from 32.8 to 41.9 % in other sites.

**Table 1:** Household and individual resident characteristics, by site (weighted analyses)

	Peru (urban)	Peru (rural)	Mexico (urban)	Mexico (rural)	China (urban)	China (rural)
*Household characteristics*						
Number of households (weighted number)	140 (703)	56 (371)	190 (600)	167 (597)	177 (508)	147 (611)
Number of residents—median (IQR)	4 (2–6)	3 (2–7)	3 (2–5)	3 (2–5)	2 (2–3)	4 (3–5)
Household composition (MV)	*2*	*0*	*3*	*0*	*2*	*1*
Older adults only (%)	14.2	28.8	33.0	30.8	39.2	12.9
Older adults with younger adults (%)	85.8	70.7	64.4	66.4	58.6	79.5
Younger adults only (%)	0.0	0.5	2.6	2.8	2.2	7.6
With children <16 years (%)	22.9	24.3	24.7	39.7	3.8	21.4
MV	2	0	3	0	2	1
Assets index—median (IQR)	9 (8–10)	8 (6–9)	8 (7–9)	6 (5–7)	8 (7–10)	9 (7–10)
MV					12	192
Home ownership (%)	91.6	96.6	97.9	100.0	52.1	100.0
MV	10	0	13	0	14	202
Agricultural land ownership (%)	0.3	17.5	6.1	25.1	0.0	58.3
MV	0	0	0	0	0	169
Any savings (%)	2.0	10.8	8.0	3.2	64.7	42.1
MV	2	0	0	0	52	201
Savings >100 % of annual HH income (%)	0	0	1.8	0.0	41.0	9.4
MV	2	0	0	0	52	201
Any debts (%)	10.0	6.1	7.1	9.3	1.8	1.9
MV	2	0	0	0	0	7
Catastrophic healthcare costs (>10 % of household income) (%)	11.4	14.2	25.2	31.1	29.1	12.2
MV	2	32	2	7	2	0
One or more indicators of economic strain (%)	42.7	78.6	54.5	46.5	10.1	25.1
MV	2	0	0	0	63	208
Overall financial situation (% bad or very bad)	4.6	15.4	18.1	15.6	2.7	8.4
MV	2	0	9	0	23	192
Overall household life satisfaction (% dissatisfied or very dissatisfied)	6.4	8.8	11.0	8.2	3.5	9.5
MV	2	0	9	0	23	192
*Individual characteristics*						
Number of individual residents (weighted)	611 (3079)	228 (1583)	685 (1980)	604 (2261)	455 (1312)	594 (2454)
Female gender (%)	58.0	50.9	64.7	54.4	59.7	50.1
MV	0	0	0	0	7	42
Age distribution—MV	*2*	*6*	*12*	*0*	*2*	*1*
<5 years (%)	5.7	3.5	2.6	2.8	0.5	2.8
5–16 years (%)	5.5	14.6	8.7	15.2	1.0	3.3
16–64 (%)	56.1	42.8	46.8	46.2	44.0	60.0
65+ (%)	32.8	39.1	41.9	35.9	54.6	33.9
Children and adults in FT education (%), by age						
5–15 years (%)	76.9 (26.0)	78.4 (14.3)	94.2	84.2	100.0	91.2
16–64 years (%)	8.1	0.0	12.7	7.4	7.0	4.0
Children and adults in paid work (%), by age						
5–15 (%)	0.0	0.0	0.0	0.9	0.0	1.2
16–64						
In work (%)	72.1	56.6	62.1	69.6	42.6	85.8
Other roles						
Seeking work (%)	2.7	0.3	1.8	1.3	8.0	0.0
Student (%)	11.5	11.9	12.5	6.6	5.2	3.6
Homemaker/child care (%)	6.1	15.6	11.7	13.8	1.5	4.1
Caring for OA (%)	3.7	3.9	1.5	1.0	0.0	3.2
Retired (%)	2.9	0.0	3.6	0.1	34.1	1.9
Limiting long-term illness or disability (%)	0.6	11.5	2.2	3.2	0.9	0.0
65+						
In work (%)	4.6	15.3	11.7	22.8	0.0	7.3
Seeking work (%)	3.3	0.0	0.0	0.0	0.0	0.0
Adults receiving a pension (%), by age						
16–64 years (%)	2.8	0.3	2.7	0.1	39.2	1.2
65+ years (%)	70.0	59.8	65.7	90.5	94.6	14.8
Monthly income from pensions (international $) for those aged 65 and over receiving a pension—mean (SD)	391 (190)	268 (75)	278 (308)	90 (122)	752 (706)	108 (125)

*MV* missing values (weighted)

Regarding economic status, household assets were fewer in rural Mexico than in other sites. Home ownership was almost universal, other than in urban China, where just over half of homes were owned. In Chinese sites, particularly in urban China, household savings were much more commonly reported and debts less so than in sites in Peru and Mexico. Negative perceptions of household economic circumstances and indicators of economic strain were also less commonly reported in the urban and rural China sites than in sites in other countries. Catastrophic healthcare spending was more common in Mexico and in urban China, than in other sites.

Children of school age were generally in full time education, with the lowest retention rates in sites in Peru. Other than in rural Mexico (0.9 %) and rural China (1.2 %) children were not engaged in paid work. Labour force participation rates for younger adults were lowest in the site in urban China (42.6 %) and highest in rural China (85.8 %). Labour force participation rates for older adults were higher in rural than urban sites; no older adults worked in the urban China site; in other sites rates varied between 4.6 and 22.8 %. Pension coverage among older adults was only 14.8 % in rural China, but in other sites ranged between 59.8 % (rural Peru) and 94.6 % (urban China). The value of pensions was notably lower in rural Mexico and rural China than in other sites, and higher in urban China.

### Household income and consumption

Median monthly equivalised household incomes varied greatly between sites, from Int$123 in rural Mexico to Int$1540 in rural China (Table 2). In Mexico and Peru household incomes were higher in urban than rural sites, but the converse was true for China. Thus, both median and mean incomes were highest in the rural China site, followed by urban China, urban Peru, rural Peru, urban Mexico and rural Mexico. Other than in rural Mexico, very few of the households met criteria for either extreme poverty (<Int$1.25/day) or poverty (<Int$2/day). Extreme poverty was noted in 7.4 % of households in rural Mexico and in no households in other sites. Poverty was noted in 26.7 % of households in rural Mexico, 0.2 % in urban Mexico, and 1.0 % in rural Peru, and in no households in other sites. The proportion of households living under the national poverty line (<60 % of median equivalised household income) was also relatively low, with the highest proportions seen in rural Mexico (49.4 %) followed by rural Peru (13.8 %), urban Mexico (3.8 %), urban China (2.3 %), rural China (0.8 %) and urban Peru (0.3 %). Variation among sites in equivalised household expenditure, and particularly, equivalised food consumption was less marked than variation in income. Median household expenditure was lower in rural than urban sites in all countries: highest in urban sites in Peru, followed by urban China, rural China, urban Mexico, rural Mexico and rural Peru. Median household expenditure was around half that of median household income in Peru and urban Mexico, and around a seventh to a quarter in China. Income and expenditure were lowest and most closely matched in rural Mexico sites. Income inequality, assessed using the 20:20 ratio was higher in rural than in urban sites in all three countries, and most marked in the sites in China, followed by those in Mexico and Peru. Inequality of consumption was much less apparent than income inequality.

**Table 2:** Monthly total equivalised household income, expenditure and food consumption (international $, 2011)—weighted analysis

Site		Equivalised household income	Equivalised household expenditure	Equivalised food consumption	Income inequality (20:20 ratio)^a^	Consumption inequality (20:20 ratio)^b^
Peru, urban n = 140	Mean (95 % CI)	838 (730–946)	321 (293–349)	163 (148–178)	3.48	2.77
	Median (IQR)	772 (557–1132)	298 (241–406)	151 (119–200)		
Peru, rural n = 56	Mean (95 % CI)	504 (358–651)	238 (161–314)	121 (93–149)	5.21	4.87
	Median (IQR)	392 (294–564)	142 (131–272)	106 (74–144)		
Mexico, urban n = 190	Mean (95 % CI)	427 (368–486)	233 (209–256)	138 (122–154)	5.47	3.54
	Median (IQR)	355 (246–487)	199 (150–299)	115 (78–172)		
Mexico, rural n = 167	Mean (95 % CI)	149 (125–173)	165 (131–200)	101 (84–117)	8.91	4.79
	Median (IQR)	123 (58–184)	143 (93–203)	92 (52–143)		
China, urban n = 177	Mean (95 % CI)	1456 (1135–1795)	284 (263–304)	182 (166–197)	8.66	3.57
	Median (IQR)	914 (694–1265)	260 (193–343)	162 (116–216)		
China, rural n = 147	Mean (95 % CI)	3128 (1828–4427)	237 (189–285)	122 (86–157)	30.17	6.48
	Median (IQR)	1540 (611–5127)	218 (123–281)	100 (46–157)		

^a^The ratio of the aggregate equivalized incomes of the top and bottom 20 % of households by income

^b^The ratio of the aggregate equivalized expenditure of the top and bottom 20 % of households by expenditure

### Variation in individual income, and income pooling by age and gender

In most sites, a high proportion of younger and older adults were reported to receive at least some income (Table 3). The exceptions were the Mexico sites, where only around one half or slightly fewer of younger adults were reported to have any source of income. The proportion of younger adults receiving an income was slightly lower for women than men in all sites, and only 36.7 % of younger women in rural Peru received an income. The proportion of older adults receiving an income was generally high, and similar for men and women, although slightly lower for women in Peru. In all sites other than urban China, median incomes, for those with an income, were higher for younger adult than older adult residents. Younger adult median incomes were lower for women than men in rural Peru and rural China but otherwise similar between genders. Older adult median incomes were higher for women than men in urban Peru, and higher for men in rural China.

**Table 3:** Individual monthly incomes for adult residents by site, age group and gender (weighted analysis)

Site	Gender	Younger adults (16–64 years)			Older adults (65 years and over)		
		Proportion with an income (%)	Median income (25th, 75th centile) for those with incomes	Proportion (%) pooling all of their income	Proportion with an income (%)	Median income (25th, 75th centile) for those with incomes	Proportion (%) pooling all of their income
Peru urban	M	87.1	706 (558–1058)	4.8	96.0	353 (292–588)	18.1
	F	70.2	765 (529–941)	8.8	89.3	588 (294–794)	20.2
Peru rural	M	83.9	529 (412–588)	0.0	88.0	282 (247–492)	1.8
	F	36.7	441 (235–471)	9.7	77.1	294 (229–353)	5.5
Mexico urban	M	52.8	410 (346–691)	18.0	99.1	277 (127–400)	14.8
	F	47.2	461 (230–691)	5.7	92.4	238 (115–388)	13.0
Mexico rural	M	44.0	114 (92–325)	20.4	94.0	58 (58–61)	30.6
	F	45.8	138 (92–259)	2.8	93.9	58 (58–128)	30.0
China urban	M	82.8	694 (412–926)	10.2	98.2	770 (694–1065)	42.8
	F	74.6	648 (509–926)	4.5	98.4	694 (527–856)	42.7
China rural	M	90.5	880 (463–7060)	26.1	94.0	370 (69–4693)	27.2
	F	87.8	521 (347–810)	25.8	97.0	191 (69–2419)	21.7

In nearly all sites the modal response to the question “does this person pool all, some, or none of their income” was ‘some’ (proportions not reported). The proportion reported as pooling all of their income was generally similar or somewhat higher (urban Peru, rural Mexico and urban China) among older adults than younger adults (Table 3). Income pooling was particularly common among older adults in rural Mexico (30 % pooling all of their income) and urban China (43 %). The propensity for older adults to pool their income did not seem to vary by gender. For younger adults, the proportion of men pooling all of their income was generally higher than that for women, other than in rural Peru and rural China.

### Sources of household income, and the relative contribution by older adults

Table 4 summarizes the distribution of the proportional contribution of different sources of income to total household income in each site. The distribution is described both in terms of the mean and the median. The means reflect the contribution of different sources to the aggregate income across all households in the site, and sum to approximately 100 % across sources (not exactly 100 % due to rounding error, and because ‘other income’ was omitted from the summary categories). However, sources of income varied greatly among households, and the distributions were markedly negatively or positively skewed for some sources in some sites. In such cases (where the median differs from the mean), the median and interquartile range provides a clearer indication of the distribution among households. In most sites other than rural China (median proportion 2 %), pensions accounted for a significant proportion of total household income. The median proportion was around one fifth in Peru, one third in Mexico and four-fifths in urban China. Income from assets, government and private transfers was negligible other than in urban Peru (private transfers), urban Mexico (government transfers) and rural China (income from assets). Thus, at site level, the mean proportional contribution of sources of income varied considerably between countries, and, between urban and rural sites. In Peru income from paid work is the main source of income, with an important contribution from pensions, and, in urban Peru, private transfers. In Mexico, pensions, followed by paid work, made the largest contribution with an important contribution from government transfers in the urban but not the rural site. In urban China, households were highly reliant upon pensions as the main source of income, with a minimal contribution from paid work. Contrastingly, in rural China, pensions made a small contribution, with income from paid work and assets dominating.

**Table 4:** Sources of household income by site; median/mean % of total household income (weighted analysis)

Site		Pension	Paid work	Assets	Gov’t transfers	Private transfers	% of total household income contributed by older adults	
							All households	Households with younger adults
Peru, urban n = 140	Mean (95 % CI)	24 (19–29)	47 (39–56)	4 (0–7)	1 (0–2)	16 (8–25)	45 (36–54)	36 (30–41)
	Median (25th, 75th centile)	22 (7–34)	52 (26–69)	0 (0–0)	0 (0–0)	8 (0–21)	37 (21–63)	33 (19–48)
Peru, rural n = 56	Mean (95 % CI)	28 (20–36)	43 (31–55)	0 (0–0)	0 (0–1)	5 (0–11)	51 (38–63)	43 (35–51)
	Median (25th, 75th centile)	20 (16–36)	52 (19–69)	0 (0–0)	0 (0–0)	0 (0–0)	40 (30–70)	40 (31–60)
Mexico, urban n = 190	Mean (95 % CI)	35 (29–40)	28 (21–36)	6 (3–10)	25 (21–29)	3 (1–5)	66 (56–75)	51 (43–59)
	Median (25th, 75th centile)	31 (12–61)	6 (0–57)	0 (0–0)	24 (9–33)	0 (0–0)	70 (31–100)	44 (26–77)
Mexico, rural n = 167	Mean (95 % CI)	52 (42–63)	36 (27–45)	1 (0–2)	3 (1–5)	2 (0–4)	66 (63–74)	53 (41–64)
	Median (25th, 75th centile)	33 (13–100)	23 (0–76)	0 (0–0)	0 (0–0)	0 (0-0)	100 (23–100)	37 (18–100)
China, urban n = 177	Mean (95 % CI)	70 (65–75)	13 (9–17)	9 (6–12)	4 (1-6)	4 (2–6)	68 (63–74)	54 (49–60)
	Median (25th, 75th centile)	82 (48–96)	0 (0–22)	3 (0-7)	0 (0–2)	0 (0–2)	76 (42–100)	52 (36–73)
China, rural n = 147	Mean (95 % CI)	12 (7–18)	41 (30–51)	45 (34–56)	1 (1–2)	1 (0–2)	34 (26–42)	27 (19–35)
	Median (25th, 75th centile)	2 (1–11)	11 (4–88)	11 (4-88)	0 (0–1)	0 (0–0)	17 (5–52)	17 (7–50)

The proportion of the total household income provided by older adult residents is summarized both for all households with an older resident, and, separately for households with both older adult and younger adult residents (Table 4). Since the income contributed by older adults is by definition 100 % for households with no younger adults, and since in all sites multigenerational households were the mode, the latter indicator may be considered to be more representative of the relative contribution of older adults and younger adults to household income. Inevitably, the proportion of the total household income provided by older adult residents is lower for multigenerational households than for all households, including those with no younger adults. Nevertheless, for multigenerational households, in Mexico, and in urban China the mean contribution of older adults was a little over one half of household income, while in rural China it was a little over one quarter. Sites in Peru were intermediate between these extremes. In one quarter of multigenerational households in rural Mexico, all of the household income was contributed by older adults.

### The impact of household pension income on labour force participation rates

For each int$100 increment in household pension income, controlling for age, gender, household assets, number of children and number of adults, younger adult residents were 2 % less likely to be in work or seeking work (pooled prevalence ratio 0.980, 95 % CI 0.965–0.996, I^2^ 0.0 %). There was also a trend towards older residents being less likely to be in work or seeking work in the context of higher household pension incomes (pooled prevalence ratio per int$100 increment 0.963, 95 % CI 0.896–1.036, I^2^ 32.7 %). Heterogeneity was accounted for by the trend in the opposite direction in the rural China site (PR 1.11, 95 % CI 0.94–1.32), and in the urban China site no older adults were working or seeking work. In the four Latin American sites, the effect of household pension income upon labour force participation of older adults was more consistent, but still not statistically significant (pooled PR 0.934, 95 % CI 0.862–1.011, I^2^ 0.0 %). The main determinants of labour force participation among older adults were male gender (pooled PR M vs F 2.87, 95 % CI 1.90–4.35, I^2^ 0.0 %) and younger age (pooled PR per one year increment in age 0.89, 95 % CI 0.86–0.92, I^2^ 15.6 %). Household wealth was not associated with older adult labour force participation (pooled PR per asset 0.98, 95 % CI 0.84–1.14, I^2^ 52.3 %) other than in the rural China site (PR per asset 0.60, 95 % CI 0.40–0.88).

### Construct validity of measures of household income and consumption

Household income was moderately positively correlated with household expenditure and household food consumption in all sites, more strongly so in urban than rural sites, and in Latin America than in China (Table 5). The same pattern of association is seen between both household income and expenditure and occupational class of the index older person. Household expenditure was uniformly strongly associated with household food consumption (from +0.79 to +0.84, by site). Both income and expenditure were modestly correlated with household assets and subjective perception of the household economic situation (economic wellbeing). The correlation between either income and expenditure, and the number of reported indicators of economic strain was not statistically significant in most sites.

**Table 5:** Correlations between equivalised household income and expenditure, and other economic indicators (non-weighted analysis)

	Peru, urban n = 139	Peru, rural n = 56	Mexico, urban N = 190	Mexico, rural n = 167	China, urban n = 171	China, rural N = 110
*Equivalised household income, and*						
Equivalised household expenditure	+0.50 (<0.001)	+0.40 (0.003)	+0.44 (<0.001)	+0.30 (<0.001)	+0.22 (0.004)	+0.25 (0.009)
Equivalised food consumption	+0.48 (<0.001)	+0.42 (0.002)	+0.44 (<0.001)	+0.26 (0.001)	+0.23 (0.003)	+0.15 (0.12)
Number of assets	+0.29 (<0.001)	+0.55 (<0.001)	+0.19 (0.008)	+0.22 (0.005)	+0.06 (0.41)	+0.35 (<0.001)
Occupational social class	+0.31 (<0.001)	+0.07 (0.63)	+0.09 (0.23)	+0.02 (0.84)	+0.22 (0.003)	+0.02 (0.85)
Economic wellbeing	+0.29 (0.001)	+0.37 (0.005)	+0.11 (0.14)	+0.18 (0.03)	+0.29 (<0.001)	+0.30 (0.001)
Number of economic strain indicators	−0.30 (<0.001	−0.34 (0.01)	−0.02 (0.73)	+0.20 (0.01)	−0.02 (0.79)	−0.31 (0.001)
*Equivalised household expenditure, and*						
Equivalised food consumption	+0.81 (<0.001)	+0.84 (<0.001	+0.83 (<0.001)	+0.79 (<0.001)	+0.82 (<0.001)	+0.81 (<0.001)
Number of assets	+0.18 (0.03)	+0.40 (0.002)	+0.23 (0.001)	+0.27 (0.001)	+0.09 (0.23)	+0.26 (0.006)
Occupational social class	+0.20 (0.20)	+0.06 (0.67)	+0.14 (0.06)	+0.02 (0.84)	+0.08 (0.29)	+0.03 (0.80)
Economic wellbeing	+0.22 (0.009)	+0.11 (0.44)	+0.08 (0.27)	+0.07 (0.41)	+0.26 (0.001)	+0.35 (<0.001)
Number of economic strain indicators	−0.12 (0.15)	−0.17 (0.22)	−0.01 (0.89)	−0.03 (0.67)	+0.24 (0.003)	−0.08 (0.43)

Relative income poverty (<60 % median household income) was more common than relative consumption poverty (<60 % median household consumption), and the agreement (Kappa) between these two categorizations was modest; +0.24 in urban Peru, +0.06 in rural Peru, +0.10 in urban Mexico, +0.46 in rural Mexico, +0.22 in urban China, and −0.02 in rural China. When assessing the pattern of independent associations of these two ways of categorizing poverty with other indicators of economic utility (Table 6), it was apparent that consumption poverty was more clearly associated with poverty of assets (number of assets and car ownership), and more consistently and robustly associated with a negative perception of the household’s economic situation.

**Table 6:** Associations of relative income and consumption poverty with other indictors of economic utility

	Peru, urban n = 139	Peru, rural n = 56	Mexico, urban N = 190	Mexico, rural n = 167	China, urban N = 171	China, rural N = 110	Pooled estimates, Higgins I^2^ (heterogeneity %)
*Associations with income poverty* ^a^							
Car ownership^b^	3.7 (0.7–20.4)	2.3 (0.7–8.4)	1.4 (0.9–2.2)	1.1 (0.3–4.5)	0.3 (0.0–2.1)	0.8 (0.5–1.4)	1.19 (0.86–1.64), 29.3 %
Number of assets^c^	1.0 (0.9–1.1)	0.8 (0.7–1.0)	1.0 (0.9–1.1)	1.1 (1.0–1.2)	1.0 (0.9–1.1)	0.9 (0.8–1.0)	0.99 (0.95–1.03), 51.5 %
Number of bedrooms^c^	0.9 (0.8–1.0)	0.6 (0.5–0.8)	0.9 (0.7–1.1)	1.2 (0.9–1.5)	0.9 (0.7–1.1)	0.8 (0.6–1.2)	0.89 (0.82–0.96), 56.0 %
Lower occupational class of index older person^d^	3.4 (1.6–7.3)	5.2 (1.0–26.6)	1.0 (0.5–2.4)	1.0 (0.4–2.7)	2.1 (0.8–5.8)	0.2 (0.0–1.6)	1.74 (1.15–2.63), 58.3 %
Number of economic strain indicators^e^	2.2 (1.1–4.3)	1.7 (0.9–3.3)	1.3 (0.8–2.1)	0.3 (0.2–0.6)	15.7 (5.5–44.8)	0.1 (0.0–0.3)	1.20 (0.90–1.60), 92.0 %
Perception of household economic situation^d^ (higher scores indicate more negative perception)	7.4 (2.2–25.5)	10.8 (0.8–146.4)	3.4 (0.9–12.2)	1.1 (0.3–3.7)	4.7 (1.5–14.6)	0.1 (0.0–0.5)	2.30 (1.33–4.01), 79.4 %
*Associations with consumption poverty* ^f^							
Car ownership^b^	Could not be estimated	Could not be estimated	0.5 (0.2–1.5)	0.1 (0.0–1.0)	1.6 (0.3–7.8)	0.4 (0.1–1.2)	0.47 (0.24–0.92), 39.3 %
Number of assets^c^	0.9 (0.8–1.0)	0.9 (0.7–1.2)	0.9 (0.8–1.0)	0.9 (0.8–1.1)	1.0 (0.9–1.1)	0.9 (0.8–1.0)	0.93 (0.89–0.98), 0.0 %
Number of bedrooms^c^	1.1 (0.9–1.2)	0.9 (0.7–1.3)	0.8 (0.6–1.1)	0.9 (0.7–1.1)	1.1 (0.9–1.3)	0.9 (0.6–1.4)	1.01 (0.92–1.10), 17.4 %
Lower occupational class of index older person^d^	4.6 (0.6–37.7)	0.2 (0.1–0.6)	4.3 (1.4–13.1)	0.7 (0.2–2.3)	1.2 (0.3–4.3)	0.2 (0.0–1.5)	Urban sites 2.72 (1.25–5.92), 20.5 % Rural sites 0.32 (0.14–0.69), 30.3 %
Number of economic strain indicators^e^	2.3 (0.9–6.0)	1.3 (0.7–2.3)	0.7 (0.3–1.5)	0.9 (0.5–1.8)	Could not be estimated	2.2 (0.8–6.2)	1.20 (0.85–1.69), 31.7 %
Perception of household economic situation^d^ (higher scores indicate more negative perception)	7.6 (1.5–36.9)	7.4 (2.7–20.7)	2.1 (0.5–9.1)	0.4 (0.1–2.1)	2.0 (0.7–5.7)	17.9 (2.7–118.6)	3.43 (1.98–5.95), 64.4 %

^a^Defined as <60 % of median equivalised household income for that site

^b^Prevalence ratio (Poisson regression)

^c^Count ratio (Poisson regression)

^d^Odds ratio (ordinal regression)

^e^Count ratio (negative binomial regression)

^f^Defined as <60 % of median equivalised household income for that site

## Discussion

In this study of older persons’ households in urban and rural catchment areas in Peru, Mexico and China, we identified high levels of household income inequality between and within sites. Much of this may be attributable to marked variation in the coverage and value of pensions. Consumption inequality was not so marked. In all sites, other than rural China, older people made an important contribution to total household income, mainly from their pension income. In settings where the contribution from older people was particularly significant, they were also more likely than others to pool their income. We were able to collect information on household income and consumption from a high proportion of participating households, and the resulting measures correlated plausibly with each other, and with other indicators of economic utility, supporting their construct validity. The main limitations of the study were the modest household sample sizes in some sites, and the low response rates in others. In urban China the low response rate was accounted for, mainly, by relocation and redevelopment around the time of the Beijing Olympics, the effect of which might be expected to be random with respect to the outcomes under study. As previously highlighted, the catchment area design limits generalisability.

Our data provide some support to both income- and consumption-based measures as valid indicators of poverty in older persons’ households in middle income country settings. While low levels of consumption are more strongly and consistently associated with other indicators of economic stress and want, it may be that a comprehensive assessment of economic utility would need to include liquid assets. Some of the large difference between income and expenditure in the China sites is likely accounted for by the relatively high savings levels. There may also have been some underestimation of expenditure or overestimation of income, although it should be noted that healthcare and home care expenditure is not included in the total expenditure index. The very high correlations in all sites between food consumption and overall household expenditure suggest that the former measure could be used as a briefer assessment tool for population survey purposes.

Clear differences persist in the economic circumstances of older person’s households between the INDEP sites and countries, reflecting the longer-term drift of national policies and the distinctive characteristics of urban and rural communities. Thus, while multigenerational extended family households are the mode in most settings, our urban China site has always stood out as having a more nuclear family structure with older people or couples living alone (Prince et al. 2008). The historic urban bias of public policy in China has led to a gross disparity in pension coverage, clearly demonstrated in the 10/66 urban and rural sites (Liu et al. 2009; Prince et al. 2008). The early retirement ages and comparative generosity of the urban cadres’ legacy and civil service pensions account for the dominance of pension income in the urban China site. However, these commitments are alleged to be effectively subsidized by new contributors to the UEPS, hence damaging the attractiveness and credibility of this scheme (Pozen 2013). Pension coverage in the rural China site has increased from 3.9 % (Prince et al. 2008) in 2003 to 14.8 %, suggesting very limited uptake of the new rural pension scheme in this district. Pension demand may be low because of the increased profits from farming activity and the recent enrichment of some farming families from land sold off expensively for construction and infrastructure projects. However, these benefits have not been evenly distributed as indicated by the highly skewed income distribution, and the high levels of income inequality. While median household income is higher than that in Beijing City, the 25th centile is lower, and consumption levels remain lower than those of the households in the urban site. In Mexico, pension coverage has remained stable at around 70 % in the urban site, but has increased from 25.4 to 90.5 % in the rural site (Prince et al. 2008), reflecting the impact of the targeted benefits provided by the ‘Oportunidades’ and ‘70 y Más’ schemes. Nevertheless, a significant minority of Mexican households continue to live in poverty, with relatively high rates of indebtedness, economic strain, and negatively perceived economic circumstances. The high rates of catastrophic health spending for these Mexican older persons’ households suggest that, for many of them, Seguro Popular has failed to deliver on its promise of financial protection alongside increased access to healthcare (Knaul et al. 2012). In the Peru sites, pension coverage has not improved since survey baseline (Prince et al. 2008), probably reflecting the delayed implementation of the Pension 65 program.

The relatively large contribution made by older residents to household incomes in older people’s households is a particularly striking finding. Given that pensions are mainly contributed by older adults, and income from paid work by younger adults, these findings are understandable in the context of the proportional contribution of these sources of income to total household income, and by extension to pension coverage, labour force participation, and the relative value of salaries and pensions. In the urban China site (where pension incomes for older adults are higher than incomes for younger adults in employment), and sites in Mexico (where the proportion of older people with an income, usually from a pension, is higher than the labour force participation rate of younger adult residents) older people and their pensions make up a particularly high proportion of household income. Of note, older people in these settings seem particularly likely to pool all of their incomes to the benefit of the wider household. This raises the question of whether provision of pensions may reduce incentives for younger adult household members to engage in paid work. No significant effect on working age adult labour force participation was noted in the evaluation of the rolling out of the 70 y Más program in Mexico (Galiani et al. 2014). We did find a small, but consistent inverse association between household pension income and labour force participation of younger adult co-residents. We cannot attribute causality to this cross-sectional association, and residual confounding is a possible alternative explanation. Furthermore, the size of the association is too small to have any real policy significance. Nevertheless, it is of potential interest when viewed through the lens of intergenerational reciprocity. Pension income may confer social protection on younger family members unable to work or find work, and support the retention of young people in secondary or tertiary education. In return independent older residents may expect to receive care and support should they need it in the future, while dependent older people may already be being cared for by younger residents who have given up work to do so. Implicit within this model is the notion that extended families may, to some extent, assort themselves within households to maximize economic utility and mutual social protection, and that provision of old age pensions may exert an influence on this process. This is one of the hypotheses to be tested in the INDEP study (Mayston et al. 2014).

Impact analyses of social pension programmes among early adopter countries suggest that the increments to household income thereby conferred play an important role in reducing indigence, and mitigating the severity of poverty (Bertranou et al. 2002; Lloyd-Sherlock 2006; Lloyd-Sherlock et al. 2012; Nyanguru 2007). A more formal, quasi-experimental evaluation of the rolling out of the 70 y Más program in Mexico presents limited evidence of small beneficial effects on diet, but not nutritional status, and mood (Salinas-Rodriguez et al. 2014). Most evaluations concur that much of the social pension tends to be pooled within the household (hence better considered as a household benefit), and that expenditure on food is prioritised, alongside investments in education and health (Barrientos 2004; Nyanguru 2007; Salinas-Rodriguez et al. 2014). Positive effects on the agency of older beneficiaries have also been noted, with enhanced participation in household decision making, and an increased sense of security and wellbeing from receiving a regular personal income (Salinas-Rodriguez et al. 2014). One objective of social pensions is to reduce the necessity for older adults to continue to engage in paid work. The introduction of the 70 y Más program in Mexico was associated with reduced participation of older beneficiaries in paid work outside the home in favour of unpaid work within the household; waged work fell by an average of 2.6 h per week, and unpaid work (mainly for family enterprises increased by 2.2 h per week (Galiani et al. 2014). Somewhat surprisingly, we did not find a particularly strong or clear effect of household pension income on labour force participation by older adults. It may be that pension levels, generally, were too low to realise this objective. In the urban China site, where pension levels were particularly high, no older adults worked. Cultural norms may also have played a role—only in rural China was asset poverty a driver of continuing labour force participation, while male gender was particularly strongly associated with older adult but not younger adult labour force participation in all sites. ‘Crowding out’ of domestic remittances (informal cash transfers from family and others) may be a more important behavioural impact of social pensions; these fell by 31 % after introduction of the 70 y Más program in Mexico ‘, almost but not entirely negating the benefit of the government cash transfer (Amuedo-Dorantes and Juarez 2013).

Although the benefits of, and necessity for social pensions are clearly established, it is also apparent that, as currently constituted, they do little to redress profound socioeconomic inequalities and unfairness arising from an unequal distribution of pension entitlements to privileged groups, stratified by labour market status. Embedding social protection within a formal labour market has been a failed strategy, to which non-contributory social pensions are a partial solution. Further efforts to integrate the working population within a properly regulated and taxed formal labour market are urgently required, particularly in the Latin American region (Bosch et al. 2013; Cecchini and Martinez 2012; Economic Commission For Latin America and the Caribbean and Development Centre of the Organisation for Economic Co-operation and Development 2012). Current policy recommendations emphasise the need to couple incentives for firms to join the formal sector (reducing the regulatory burden, entry costs, and marginal taxation rates, and increasing the benefits of formal status) with legislation, enforcement and anti-corruption drives (Bacchetta et al. 2009). Workers can be encouraged and enabled to enter the formal labour market through better education, and portable skills training. Market reforms need to be introduced sensitively, since, although free trade and export-driven economies promote the development of large enterprises in the formal sector, there can be short term negative effects on both formal and informal sectors (Bacchetta et al. 2009). Future fiscal sustainability also depends upon transition towards properly funded “pay-as-you-go” contributory pension schemes, meaning that contributions from current members need to be protected against unfunded legacy commitments (Pozen 2013). Fortunately, current and future levels of economic growth, boosted by the demographic dividend, should be sufficient for countries such as Peru, Mexico and China to complete these transitions, whilst incrementally boosting investment in non-contributory schemes, both to extend coverage, and raise the basic minimum income level for older citizens (Cecchini and Martinez 2012; Gan 2013; Pozen 2013; Wang et al. 2014). Such policies would have a profound effect on reducing inequalities; for example, in China, where the Gini coefficient has recently been re-estimated at 0.61 (compared with a global average of 0.44), the difference in coverage and security level provided by retirement insurance and pension accounts accounted for a remarkable 25 % of the gap between rural and urban household incomes (Gan 2013). Social investment in Latin American countries increased by five percentage points to 18 % of GDP from 1990 to 2008, with positive impacts on income inequality in countries such as Argentina, Brazil, Chile, Costa Rica and Uruguay (Cecchini and Martinez 2012; Gasparini et al. 2010). Nevertheless this lags behind the norm for Organisation for Economic Co-operation and Development countries (25 % of GDP), and Peru and Mexico stand out as countries whose social investment is much less than would be expected from their development status (Cecchini and Martinez 2012). Underinvestment in government transfers is even more marked in China, where just 12.3 % of public fiscal expenditure is on social welfare (Gan 2013).

## Conclusions

In conclusion, despite recent improvements in social protection in two of the three countries studied, the introduction of new policies including social pensions, targeted cash transfers and health insurance programs have, so far, had little impact on the households where older people live in the 10/66 INDEP study catchment area sites. In these settings, prospects in old age are still starkly stratified by urban and rural residence, and by the benefits conferred by occupational history, where comparatively generous entitlements linked to formal jobs were extended to a small elite.

## Methods

### Settings

The INDEP study is conducted in 10/66 Dementia Research Group survey catchment areas in four countries; China, Peru, Mexico and Nigeria (Mayston et al. 2014). The analyses presented in this paper include INDEP data collected from the urban and rural sites in Peru, Mexico and China. Ethical approval for these studies was provided by King’s College London Research Ethics Committee, and by the relevant Institutional Review Boards in Peru, Mexico and China. Participation was on the basis of informed, signed consent. The Peru sites comprising urban catchment area sites (1381 older people sampled at baseline in Lima Cercado and San Miguel in the capital city, Lima) and rural sites (n = 552 in Cerro Azul, Imperial, Nuevo Imperial, Quilmana, San Luis, and San Vicente in Canete coastal province). In Mexico we also sampled urban (n = 1003 in six districts in Tlalpan, Mexico City) and rural sites (n = 1000 in nine villages in Morelos, a mountainous district 70 km from Mexico City). The urban site in China was Xicheng, close to Tiananmen Square in Beijing City (n = 1160), while the rural site comprised 14 villages in Daxing, a rural district 40 kilometres away (n = 1002). The catchment area sites are not nationally representative, nor even necessarily representative of the city or rural region where they are located. Urban areas were selected to be predominately lower socioeconomic status, or mixed neighborhoods, avoiding middle class or professional enclaves (Prince et al. 2007). Rural areas were selected to be distant from conurbations, and to include a high proportion of inhabitants with agrarian occupations.

### Economic development and social protection in the INDEP study sites

Over the ten years since the sites were originally selected (2003–2006) several have undergone significant change and development. This is most evident for the rural China site, where in the context of national economic reforms, land ownership has been granted, and agricultural land has been sold off for property development and infrastructure projects. Beijing’s second airport is soon to be constructed in the district. The urban China site, in the heart of Beijing city, has been affected by construction projects linked to the Beijing Olympics, accounting for the difficulty in tracing participants from previous waves of the 10/66 survey. The rural Peru site was severely affected by an earthquake in August 2007, and reconstruction is still under way.

All three countries have benefited from sustained high levels of economic growth over the last decade, around 4–6 % per annum in Mexico and Peru, and 8–10 % per annum in China. Economic growth has enabled countries across Latin America to pay down national debt while investing more in social protection. China, too, has made great strides in improving pension coverage and reforming healthcare finance (Pozen 2013). Nevertheless, in all three countries, the fragmented architecture of pension and social assistance programs has resulted in gaps in coverage, inequality and unfairness (Bosch et al. 2013; Rofman and Oliveri 2012; Wang et al. 2014). In Latin America generally, coverage by contributory pension schemes is low because of the ceiling imposed by the dominant informal labour sector; coverage by contributory pensions, around 40 %, has been boosted to 60 % by the recent rise in non-contributory pension schemes, but these are generally of much lower value (Bosch et al. 2013; Rofman and Oliveri 2012). In Mexico City, a universal social pension (currently $65 per month) was introduced for all those aged 70 and over in 2001. The federal system of conditional cash transfers (‘Oportunidades’) provides around $22 per month for older people among the poorest families. A federal system of social pensions ‘70 y más’ has been rolled out state by state starting with rural communities (population <2500) from 2007, and extended in 2011 to communities with a population of less than 30,000. With the election of the Institutional Revolutionary Party in 2012, this program will be extended to some poor urban neighborhoods and to those aged 65 years and over. The 500 Mexican Pesos ($34) per month provided to the beneficiaries of the 70 y más program can be compared with the average of 17,500 Mexican pesos ($1174) per month for beneficiaries of some contributory schemes. In Peru, as with Mexico, the formal labour sector is smaller than the Latin American average, and pension coverage has historically been among the lowest in Latin America (Rofman and Oliveri 2012). After the last election the government of President Humala committed to the introduction of means tested pensions of $40 a month (Pension 65) for all those with no contributory pension entitlement. In China there are essentially four types of pension scheme (Pozen 2013; Wang et al. 2014); (1) before 1997, state-owned enterprises (SOEs) provided non-contributory pensions, now referred to as ‘legacy pensions’—this then evolved into a contributory Urban Enterprise Pension System (UEPS) covering 280 million urban workers, mainly employees of large private enterprises and State-Owned Enterprises; (2) the Rural Pension social endowment scheme, rolled out from 2009 to 2012 allows rural workers to make voluntary contributions to individual accounts that are subsidized by local and central government—those aged 60 and over at inception of the scheme could still benefit without contributions, so long as their children chose to contribute; (3) from 2011, a similar old age insurance scheme was available for urban residents not eligible for the UEPS; and (4) a non-contributory pension for civil servants, now being reformed towards a contributory system with 8 % salary contributions from employees.

### Sampling

For each site, we sampled from among those households where one or more older participants had been interviewed at baseline and follow-up, categorizing these households as follows.

1. Incident care households (where all older residents were independent at baseline, but in which one or more have become care dependent by the incidence survey.
2. Chronic care households (with one or more care dependent older people at baseline, who remained care dependent in the incidence survey).
3. Control households (where all older residents were independent at baseline, and remained so at the incidence survey).

All households meeting criteria for incident or chronic care were selected for inclusion in the INDEP study. In each site, control households equivalent in number to the sum of incident and chronic care households were selected in each site, at random from all those eligible, and batch matched to care households for the age of the oldest resident.

### Data collection

For each selected household, we aimed to conduct a household interview with a suitably qualified key informant (usually the self-defined head of household), brief interviews with each of the surviving index older people, and an informant interview for each older person to provide an independent perspective on their health and needs for care. The household interviews were conducted masked to the household group status.

### Measures

A full account of the interviews administered in the INDEP study is provided in our open access protocol paper (Mayston et al. 2014). Here we summarise only those elements used for the analyses presented in this paper. Household interviews for household income and consumption have not been used in previous waves of the 10/66 survey. The questions for the INDEP study were developed from questionnaires used successfully in community research into social pensions, poverty and wellbeing in South Africa and Brazil (Lloyd-Sherlock et al. 2012). We checked in a preparatory meeting with local investigators the relevance and comprehensiveness of questions regarding sources of income and types of expenditure, and adjusted the phrasing of questions for each country to reflect local systems. The detailed household interview comprises:

1. Household composition and roles—the ages, genders, marital, educational and occupational status of all residents. For the purposes of the analyses presented in this paper those aged 0–15 years are referred to as children, those aged 16–64 years as younger adults, and those aged 65 and over as older adults.
2. Economic evaluation.
  - A household assets index covering household goods and amenities (telephone or mobile phone, stove, electricity supply, television, radio or stereo, refrigerator, sewing machine, bicycle, computer, and motor vehicles), and ownership of land, property and livestock.
  - Assets in savings or investments (bank or savings account, stocks or shares).
  - Monthly household income was estimated by enquiring systematically about 20 different sources of income and allocating each to an individual resident, or to the household if not specifiable. Income sources were clustered into five groups; pensions (government social pensions, employer pension or retirement annuity), paid work (full or part-time regular or occasional work, or income from a business, and any employment benefits), income from assets (savings, investments, property rents, lodgers), government transfers (unemployment benefit, child support grants, disability benefits, public work schemes) and private transfers (money from religious organisations, non-governmental organizations (NGOs) or charities, gifts or regular payments from family or others outside of the household). Total monthly household income was calculated by summing after tax income across all sources and all residents. This monthly amount was then equivalised by dividing by the modified Organisation for Economic Cooperation and Development (OECD) equivalence scale (1.0 for the first adult, 0.5 for all other adults, and 0.3 for children) to account for economies of scale and converted into 2011 international dollars using purchasing power parity (PPP) exchange rates (The World Bank 2014). This approach allows us to estimate total household income and income for each household member, by source.
  - Consumption, 25 items eliciting food consumption (the value or cost of all food consumed at home and outside of the home), household expenses and other personal expenditure, also divided by the OECD equivalence scale. Consistent with convention, health and social care expenses were not included in general consumption, but catastrophic healthcare costs were flagged, separately, when households had spent more than 10 % of household income in the last three months on health care.
  - Household debt and loans, and other indicators of financial strain. These included; asking for help from friends or relatives, an employer, a religious organisation, or charity; borrowing from a bank, moneylender or loan shark; cutting down on food consumption; trying to find extra work; running up an account with a shop; applying for a grant; apply for food parcels or vouchers; drawing on savings, selling stocks or shares; any other action to address the financial difficulty.
  - Subjective assessment of overall financial status; How would you rate the financial situation of this household at present? Is it very good, good, average, bad or very bad?

### Analyses

1. Descriptive analyses, by site, weighted to take account of sampling fractions of care and control households, and non-response at household level, aiming for generalizability to the incidence phase of the 10/66 surveys in each catchment area site (Mayston et al. 2014; Prince et al. 2007). We summarize.
  - Individual characteristics, the age and gender distribution of all residents, the pension coverage and employment rate among adults,
  - Household characteristics; household composition, assets, home and agricultural land ownership, savings, debts, economic strain, catastrophic healthcare spending, subjective assessment of household financial situation, and life satisfaction.
  - Mean and median total household equivalised income, expenditure and food consumption.
  - As a measure of local income inequality, the 20:20 ratio, defined as the ratio of the income of the highest 20 % of households to that of the lowest 20 %, an analogous calculation performed for consumption inequality.
  - The prevalence of absolute poverty (extreme poverty <$1.25 per day, and poverty <$2 per day) and relative poverty below the national ‘poverty line’ (equivalised household income <60 % of the national median^1^).
  - The proportion of individuals with an income, and median income for those receiving an income stratified by gender and age group (younger adults aged 16–64 years, and older adults aged 65 years and over). For those receiving an income we also report the proportion reporting that they pooled all of their income within the household.
  - The mean and median composition (%) of household income by source (pension, paid work, assets, government and private transfers).
  - The proportion of total household income contributed by older adults in all households with an older adult resident, and in the subset of multigenerational households comprising one or more older adults living with younger adults.
2. Mixed effects Poisson regression (individual residents nested within households), was used to estimate the effect (prevalence ratio) of household pension income on labour force participation, separately for older adult and younger adult residents, controlling for likely confounders (the main hypothesized determinants of individual labour force participation) resident age and gender, household size and composition (number of child and adult residents) and household assets.
3. Construct validity.
  - Spearman non-parametric correlations between equivalised household income and expenditure, and equivalised food consumption, assets, occupational social class (index older person), perception of household economic situation, and economic strain indicators.
  - The associations between relative income poverty (<60 % of site-specific median equivalised household income) and consumption poverty (<60 % of site-specific median equivalised household expenditure), and car ownership (estimated using Poisson regression, prevalence ratio); number of assets, number of bedrooms (Poisson regression, count ratio); economic strain indicators (negative binomial regression, count ratio), perception of household economic situation, occupational class of index older person (ordinal regression, odds ratio). Site specific associations were pooled using fixed effects meta-analyses, with Higgin’s I^2^ to reflect heterogeneity among sites.

## Abbreviations

Int$: international dollars
NGO: non-governmental organization
OECD: Organisation for Economic Cooperation and Development (OECD)
PPP: purchasing power parity
UEPS: Urban Enterprise Pension System

## Footnotes

1 National median disposable household income data was obtained from Euromonitor (Peru), National Institute of Statistics and Geography—INEGI (Mexico) and the China Household Finance Survey (China). Where necessary, equivalisation factors were applied using data collected in our surveys. The 60 % poverty line was adjusted up to 70.6 % to account for a likely 15 % underestimation of income by these national survey sources. The resulting poverty lines (monthly equivalised household income) were—Peru int$231.4, Mexico int$118.4 and China int$178.3.
